# RETRACTED: Abo Mansour et al. The Potential Neuroprotective Effect of Thymoquinone on Scopolamine-Induced In Vivo Alzheimer’s Disease-like Condition: Mechanistic Insights. *Molecules* 2023, *28*, 6566

**DOI:** 10.3390/molecules30224436

**Published:** 2025-11-17

**Authors:** Hend E. Abo Mansour, Aya Ibrahim Elberri, Mai El-Sayed Ghoneim, Waad A. Samman, Aisha A. Alhaddad, Mahmoud S. Abdallah, Eman I. El-Berri, Mohamed A. Salem, Esraa M. Mosalam

**Affiliations:** 1 Biochemistry Department, Faculty of Pharmacy, Menoufia University, Shibin El-Kom 32511, Egypt; 2 Genetic Engineering and Molecular Biology Division, Department of Zoology, Faculty of Science, Menoufia University, Shibin El-Kom 32511, Egypt; 3 Department of Pharmacology and Toxicology, Faculty of Pharmacy, University of Sadat City (USC), Sadat City 32897, Egypt; 4 Department of Pharmacology and Toxicology, College of Pharmacy, Taibah University, Medina 42353, Saudi Arabia; 5 Clinical Pharmacy Department, Faculty of Pharmacy, University of Sadat City (USC), Sadat City 32897, Egypt; 6 Clinical Pharmacy Department, Faculty of Pharmacy, Tanta University, Tanta 31527, Egypt; 7 Department of Pharmacognosy, Faculty of Pharmacy, Menoufia University, Shibin El-Kom 32511, Egypt

The journal retracts the article “The Potential Neuroprotective Effect of Thymoquinone on Scopolamine-Induced In Vivo Alzheimer’s Disease-like Condition: Mechanistic Insights” [1] cited above.

Following publication, concerns were brought to the attention of the Editorial Office regarding the integrity of a number of images presented in this publication [1].

Adhering to our standard procedure, an investigation was conducted by the Editorial Office and Editorial Board, which identified inappropriate image editing and partial duplication in Figures 2 and 4 presented within this article. Consequently, the Editorial Board has lost confidence in the reliability of the findings and has decided to retract this publication, as per MDPI’s retraction policy (https://www.mdpi.com/ethics#bookmark30).

This retraction was approved by the Editor-in-Chief of the journal *Molecules*. Although the authors corroborated with supplementary materials in response to this investigation, it did not meet the Editorial Board’s expectations of resolving these concerns. The authors did not agree to this retraction.
